# Daily Negative Affect and Reaction Time Inconsistency in Emerging Adults: Ecological Momentary Assessment Study

**DOI:** 10.2196/64397

**Published:** 2026-06-09

**Authors:** Lauren Alexandra Rutter, Pei-Ying Chen, Prabhvir Lakhan, Laura Thi Germine, Zoe Hawks

**Affiliations:** 1 Department of Psychological and Brain Sciences, Indiana University, 1101 E 10th Street, Bloomington, IN, 47405, United States, 1 (812) 856-9953; 2 Digital Scholarship & Sciences, University of Nebraska-Lincoln Libraries, Lincoln, NE, United States; 3 Division of Brain and Cognitive Health Technology, Department of Neurolgy, Albert Einstein College of Medicine, Bronx, NY, United States; 4 Brooklyn Health, Brooklyn, NY, United States

**Keywords:** ecological momentary assessment, negative affect, cognition, emotions, questionnaire, neuroticism, anxiety, depression, insomnia, stress

## Abstract

**Background:**

Anxiety and mood disorders, characterized by elevated negative affect (NA) and cognitive impairments, are highly prevalent among college students. Within-person (WP) NA variability, which captures moment-to-moment fluctuations in NA, provides unique insights into emotional processes that are not reflected in mean NA levels. Cognitive variability, particularly reaction time (RT) inconsistency, is increasingly recognized as a sensitive marker of cognitive health and functional integrity. Although prior research links NA to cognitive variability, the short-term dynamics of these associations in naturalistic settings remain understudied. College students provide an ideal population for examining these dynamics using ecological momentary assessment (EMA).

**Objective:**

This study investigated the association between WP NA and RT inconsistency, hypothesizing that higher WP fluctuations in NA would predict increased RT inconsistency. We also examined the moderating roles of practice effects and covariates, including neuroticism, insomnia, and sex.

**Methods:**

Using EMA, 99 university students completed morning and evening assessments over 14 days, including a cognitive task measuring RT inconsistency (standard deviation in trial-level RT) and self-reported NA. Multilevel modeling was used to assess WP fluctuations in NA and their impact on RT inconsistency, accounting for time (session number), between-person differences in NA, and covariates such as sleep problems, neuroticism, age, sex, and use of a touch device.

**Results:**

WP fluctuations in NA significantly predicted increased RT inconsistency (exp(β)=1.022, 95% CI 1.008‐1.037; *P*=.007), supporting the hypothesis that NA variability disrupts cognitive performance. Male students exhibited lower RT inconsistency than female students, with a small effect size (exp(β)=0.824, 95% CI 0.694‐0.977; *P*=.049). Finally, EMA sessions were inversely associated with RT inconsistency, with a stronger effect up to session 3 (exp(β)=0.930, 95% CI 0.879‐0.985; *P*=.03) than after session 3 (exp(β)=0.986, 95% CI=0.979‐0.992; *P*<.001), indicating practice effects.

**Conclusions:**

Momentary fluctuations in NA influence cognitive variability, particularly in the early stages of repeated cognitive tasks, underscoring the role of emotional processes in cognitive performance. Practice effects and individual differences, such as sex and insomnia, influence these associations. These findings highlight the use of EMA for understanding cognitive-affective processes and suggest potential intervention targets, such as addressing NA, to improve cognitive functioning in emotionally vulnerable populations like college students.

## Introduction

Anxiety and mood disorders, also known as emotional disorders, are the most common mental disorders and leading contributors to the global burden of disability [1,2]. Emotional disorders are often chronic and highly comorbid due to their shared features, including a high level of negative affect (NA) and strong reactivity to NA, also known as neuroticism [3]. Elevated NA is typically accompanied by both affective dysregulation and other cognitive impairments such as lower executive functioning, reduced attention capacity and processing speed, and impaired short-term memory, all of which are associated with the severity of emotional disorders and the likelihood of relapse [4,5]. NA has been extensively linked to emotional disorders, yet its momentary fluctuations and their cognitive implications remain underexplored. This study bridges this gap by using ecological momentary assessment (EMA) to examine the real-time interplay between NA and cognitive variability.

Recent research using EMA has examined within-person (WP) variation in NA. Broadly, this body of work demonstrates that dynamic fluctuations in NA are associated with increased psychiatric symptoms [6-9]. Emotional inertia, or the tendency for emotional states to persist over time, represents another critical dimension of affective dynamics [10]. Prolonged negative emotional states may indicate difficulties in emotional recovery, contrasting with high emotional instability, which reflects rapid fluctuations in affect. Both extremes of these dynamics are linked to the risk of mood disorders [11]. EMA allows researchers to measure both affect inertia and affect fluctuations in real-time, offering a nuanced perspective on these emotional processes and their cognitive implications. This study focuses on momentary WP fluctuations in NA, which have been consistently linked to psychiatric symptoms. In prior EMA research, in a study of 40 individuals with depression and 40 healthy controls, participants with depression exhibited more variable NA [8]. In another EMA study (N=365), participants with a current depression and/or anxiety disorder had higher NA fluctuations, suggesting sensitivity to internal and external sources of stress coupled with poor emotion regulation skills [11]. Based on a meta-analysis of 79 studies on emotion dynamics and psychological well-being, low psychological well-being tends to occur when there are more variable and unstable emotions, especially negative emotions [7].

NA is consistently linked to cognition, but the mechanisms accounting for these relationships remain undercharacterized and poorly understood [12]. One theory is that the associations between NA and cognitive ability may reflect the underlying integrity of the nervous system and the efficiency of information processing [12]. Efficient information processing refers to the ability to quickly and accurately process stimuli and integrate them with appropriate responses. Furthermore, efficient information processing could explain why better cognitive ability is related to better physical and mental health [12]. However, the naturalistic, short-term (eg, day-to-day) time course of WP associations between NA and cognition remains unclear. This study integrates Barlow et al’s [3] framework on neuroticism and affective variability and Jokela’s [12] theory on neural efficiency to establish a unified understanding of how fluctuations in NA influence cognitive performance. Reaction time (RT) inconsistency serves as a sensitive indicator of these cognitive-affective dynamics, reflecting momentary fluctuations in attention and processing speed. By using EMA, we aim to elucidate the real-world implications of these theoretical constructs.

WP variation in cognition has been discussed in the literature in several ways, including (1) variability across tasks measured at a single time point, known as dispersion; (2) variability across trials within a single task, known as inconsistency; and (3) variability over time across repeated task administrations, referred to as fluctuations [13]. Cognitive dispersion and inconsistency have been shown to increase with age and may serve as important indicators of cognitive functioning across the lifespan [14-16]. In comparison to the number of studies on variation in cognition in older adults, much less is known about physical and mental health-related correlates of WP variation in cognition in emerging adults. Our study targets WP variation in cognition in college-age students using RT-based measurement, specifically, standard deviation in trial-level RTs. Standard deviation in RT, which we refer to as “RT inconsistency,” is increasingly accruing evidence as an indicator of cognitive health, which also includes domains of executive functioning, attention, and memory [17]. RT inconsistency has potential for furthering our understanding of dynamic processes in health, cognition, and affect [18,19]. As a WP metric that can be readily assessed using EMA, RT inconsistency may yield novel insights in emerging adults who are experiencing stress and NA in their daily lives. Moreover, RT inconsistency, rather than mean RT, can represent a dimension of cognitive performance that is a dispositional characteristic reflecting underlying changes in psychological processes or neurological functioning [19]. However, while RT inconsistency is a valuable indicator of cognitive health, it is also important to study other factors, such as sleep, fatigue, and other learned behaviors. Our study controls for baseline insomnia and neuroticism in assessing the dynamic relationship between RT inconsistency and NA variability.

College students face significant academic, social, and personal stressors, including high levels of academic pressure, the transition to independent living, and the challenge of navigating new social environments [20]. These stressors contribute to emotional dysregulation, particularly fluctuations in NA, which can impair cognitive functioning such as attention and memory [21]. Furthermore, college students are a population with high rates of emotional disorders and treatment-seeking behaviors [20], making them an ideal group for studying the dynamic interplay between NA and cognition. These developmental transitions, along with the increased prevalence of anxiety and mood disorders, underscore the importance of understanding how fluctuations in affect influence cognitive performance during this critical period of young adulthood [22]. In a study of older and young adults who reported on daily stress and completed daily tasks of cognitive performance, WP variability in stress predicted cognitive fluctuations, evidenced by WP RT variability on a 2-back working memory task in both groups [23]. This is consistent with the attention-depletion hypothesis, which posits that stress-related cognitive interference disrupts attentional resources, weakening attention-dependent cognitive processing [23,24].

While the impact of stress on college students, the impact of NA on higher-level cognition [25,26], and the associations among stress, NA, and cognition in individuals with emotional disorders (see review by Pechtel and Pizzagalli [27]) have been well-established, there remains a surprising lack of published research using EMA to understand dynamic, short-term, WP connections among these factors. A better understanding of the mechanisms that connect momentary changes in affect and momentary changes in cognition is now possible through EMA. Specifically, a better understanding of RT inconsistency and its relationship with NA may advance our understanding of psychological functioning and cognitive health [13,17]. Understanding the relationships between cognition, NA, and emotional disorder symptoms in college-age students and emerging adults could aid in providing targeted directions for treatments of individuals vulnerable to stress, NA, and disrupted cognitive performance.

In summary, symptoms of emotional distress are associated with elevated NA [3,28], greater NA variability and instability [29-31], and poorer cognitive performance [32]. However, NA and cognition are not typically measured together in high-frequency study designs, which obscures insights into the momentary effects of NA on cognition. This study addressed this gap by examining WP associations between NA and RT inconsistency in university students. Participants completed daily EMAs measuring NA and cognition. For each EMA, RT inconsistency was operationalized as the standard deviation in trial-level RTs [15,33,34]. RT inconsistency was chosen as the primary measure due to its sensitivity to moment-to-moment fluctuations in cognitive processing. Compared to other measures, such as working memory or attention switching, RT inconsistency captures variability in attentional control and neural efficiency [12], making it particularly relevant for studying the cognitive impact of NA.

Using a 14-day EMA design, we examined WP associations between NA and RT inconsistency in 99 university students. We hypothesized that momentary WP increases in NA would be associated with greater RT inconsistency at subsequent assessments. As a secondary hypothesis, we examined whether individual differences in sex, neuroticism, and insomnia severity were associated with RT inconsistency. Examining these processes in daily life may clarify mechanisms linking emotional distress to functional impairment.

## Methods

### Participants

Participants were sampled from (1) a psychology student subject pool at a large Midwestern university (n=78) and (2) a paid study in which individuals were recruited through university online classified advertisements and flyers posted around campus (n=72). Inclusion criteria included being a university student aged 18 years or older with an email address, access to the internet, and a device to complete study assessments. Exclusion criteria included individuals younger than 18 years.

### Ethical Considerations

This study was reviewed and approved by the Indiana University Institutional Review Board as human subjects research (protocol numbers 2011722518 and 12537). All study procedures complied with institutional and federal guidelines for the ethical conduct of research involving human participants. Informed consent was obtained electronically from all participants prior to study enrollment. Participants were informed that participation was voluntary and that they could withdraw from the study at any time without penalty. Informed consent covered both the baseline assessment and the subsequent 14-day EMA protocol. To protect the privacy and confidentiality of participants, all data were deidentified. Identifying data (email addresses used for study communication) were stored separately from survey and cognitive task data on secure servers accessible only to the research team via 2-factor authentication. Participants recruited through the psychology subject pool received course credit for participation. Participants recruited through paid advertisements were compensated up to US $60 for completing at least 75% (18/24) of EMA assessments. No additional incentives were provided.

### Procedure

The 14-day EMA protocol was chosen to capture meaningful WP variability while maintaining participant retention, as longer durations may lead to increased dropout rates and data loss [35]. The frequency of daily assessments (2‐13 d) was selected to balance participant burden with the need to capture fluctuations in NA and cognitive performance. This schedule ensures adequate data collection while minimizing participant fatigue, which could compromise the quality of responses. The morning and evening sessions are consistent with prior EMA studies [23], and they allow us to assess affective and cognitive fluctuations at multiple time points.

Participants completed all study assessments on their personal devices. They were prompted to complete EMAs twice daily via email. We assessed for technical issues after each EMA and explored device compatibility in our prior work [36]. All assessments for the study were completed via Qualtrics and TestMyBrain.org. TestMyBrain.org is a citizen science research initiative that allows people to participate in studies in exchange for individualized feedback on their performance or self-reported characteristics [36-38]. Data from TestMyBrain.org have been shown to be of similar high quality to data gathered in a laboratory setting [37]. On day 1 (baseline), participants were emailed at 7 AM to complete their baseline assessment battery, and they were sent a reminder email at 5 PM to complete their day 1 battery by midnight. They were advised to complete the assessment battery when they had approximately 45 minutes of uninterrupted time, specifically, “in 1 sitting, without distraction.” On the following days, participants received daily links to their email addresses to complete study assessments in the morning and evening each day. Morning study reminders were sent at 7 AM and evening study reminders were sent at 4 PM. Participants were reminded to complete EMAs on days 2 to 13 when they had 5 minutes of uninterrupted time. On day 14 (termination), participants were sent links to complete their assessment battery at 7 AM, with a reminder at 5 PM to complete the assessments before midnight. They were advised to complete the battery when they had approximately 35 minutes of uninterrupted time. When participants missed more than 2 days in a row of tests, they were reminded by email to complete as many assessments as possible. Participants were compensated, either by course credit or electronic gift cards, approximately 2 days following the study termination.

### Measures

This study is part of a larger study measuring cognition and emotions over time. Relevant assessments for this study are included in Textbox 1.

Textbox 1. **Schedule of ecological momentary assessments.**

**Ecological momentary assessment days 1‐14**

- Day 1 (baseline)
  - Demographic questionnaire
  - Depression, Anxiety, and Stress (DASS)
  - Generalized Anxiety Disorder-7 (GAD-7)
  - Positive and Negative Affect Schedule—Short Form (PANAS-SF)
  - Multidimensional Emotional Disorder Inventory (MEDI)
  - Insomnia Severity Index (ISI)
  - Choice Reaction Time (Choice RT)
- Days 2‐13
  - Morning (7 AM-12 PM)
    - PANAS-SF
    - Choice RT
  - Evening (4 PM-12 AM)
    - PANAS-SF
- Day 14
  - DASS
  - GAD-7
  - PANAS-SF
  - MEDI
  - Choice RT

#### Positive Affect and Negative Affect Schedule—Short Form (PANAS-SF)

The PANAS-SF is a widely used and extensively validated 20-item self-report scale assessing positive affect (PA) and NA [39]. Items are rated on a 5-point Likert scale ranging from 1 (“Very slightly or not at all”) to 5 (“Extremely”), yielding scores from 10 to 50 for PA and 10 to 50 for NA. Higher scores indicate higher levels of affect for both subscales. The PANAS sampling schedule is described in Table 1. On day 1, participants were instructed to complete the PANAS based on how they felt in the past week. On days 2 to 14, participants were instructed to complete the PANAS based on how they felt at the time of EMA (ie, “right now”). In our sample, internal consistency was acceptable (Cronbach α=0.75).

**Table 1.** Sample characteristics of participants who completed any ecological momentary assessments after baseline (day 1).

Variable	More than 50% (n=99)	Less than 50% (n=17)	*P* value^a^
Sample, n (%)			.10
Student	37 (37)	10 (59)	
Paid	62 (63)	7 (41)	
Age (y), mean (SD; range)	22.9 (7; 18.0-56.0)	21.9 (7.3; 18.0-47.0)	.07
Sex, n (%)	74 (75)	9 (53)	.08
Neuroticism^b^, mean (SD; range)	16 (8; 1-33)	15 (9; 0-29)	.6
DASS-Depression^c^, mean (SD; range)	9 (9; 0-38)	10 (11; 0-40)	.9
DASS-Anxiety^d^, mean (SD; range)	8 (7; 0-38)	6 (8; 0-30)	.2
GAD-7^e^ total, mean (SD; range)	6.2 (4.9; 0.0-21.0)	5.5 (4.7; 0.0-19.0)	.6

a Pearson chi-square test, Wilcoxon rank-sum test, and Fisher exact test.

b Multidimensional Emotional Disorder Inventory Neuroticism scale.

c DASS-Depression: Depression Anxiety and Stress Scale—Depression subscale.

d DASS-Anxiety: Depression Anxiety and Stress Scale—Anxiety subscale.

e GAD-7: Generalized Anxiety Disorder-7.

#### Insomnia Severity Index

The Insomnia Severity Index is a 7-item self-report scale validated for measuring the insomnia symptom severity over the previous 2 weeks [40-42]. Items are rated on a 5-point Likert scale ranging from 0 (“none”) to 4 (“very severe”), yielding total scores of 0 to 28. Total scores are interpreted such that 0 to 7 indicates the absence of insomnia, 8 to 14 indicates subthreshold insomnia, 15 to 21 indicates moderate insomnia, and 22 to 28 indicates severe insomnia. In the sample, internal consistency was good (Cronbach α=0.88).

#### Depression Anxiety and Stress Scales

The Depression Anxiety and Stress Scales (DASS) is a 21-item measure rated on a 4-point Likert scale from 0 (“does not apply to me at all”) to 3 (“applied to me very much, or most of the time”) [43]. It assesses levels of depression, generalized anxiety, and general NA or stress. Based on factor analytic work, the DASS demonstrates excellent psychometric properties [42,43]. In our sample, internal consistency was good (Cronbach α=0.89).

#### Multidimensional Emotional Disorder Inventory

The Multidimensional Emotional Disorder Inventory (MEDI) assesses transdiagnostic dimensions of emotional disorder psychopathology [44]. It consists of 49 items that are rated on a 9-point Likert scale from 0 (“Not characteristic of me/does not apply to me”) to 8 (“Extremely characteristic of me/applies to me very much”). The initial validation of the MEDI used factor analysis to determine the factor structure of 9 empirically supported transdiagnostic dimensions proposed by Rosellini and Brown [44]. These 9 dimensions include neurotic temperament, positive temperament, depression, autonomic arousal, somatic anxiety, social anxiety, intrusive cognitions, traumatic re-experiencing, and avoidance. The MEDI showed strong evidence of reliability and validity in its initial psychometric evaluation in clinical and outpatient samples [43]. We used the MEDI neuroticism subscale as our measure of neuroticism, with higher scores indicating a higher degree of neuroticism. In our sample, internal consistency was excellent (Cronbach α=0.94).

#### TestMyBrain Choice RT

TestMyBrain choice RT measures psychomotor response speed, response selection, and cognitive inhibition [45,46]. It was adapted for brief digital administration from Maljkovic and Nakayama [47] and demonstrates high between-person (BP) and WP reliability in EMA [48]. Each administration lasted approximately 2.5 minutes and included 4 practice trials followed by 24 task trials. On each trial, participants are presented with 3 vertically aligned squares (blue or yellow) containing an arrow pointing either left or right. One of the 3 squares is always of a different color from the other 2, and participants are asked to choose the direction of the arrow in the differently colored square as quickly as possible by clicking or tapping their touchscreen. Participants had 5000 ms to respond to each trial, with an intertrial interval of 700 to 1500 ms. RT inconsistency was computed for each EMA as the standard deviation of trial-level RTs.

### Data Cleaning

EMA data cleaning aimed to maximize the signal-to-noise ratio by excluding data that were strongly suggestive of careless responding. From our full sample of 150 individuals, we first excluded participants who failed to complete EMAs after the baseline assessment (day 1). The remaining sample included 116 participants. Second, we excluded participants who failed to complete the morning EMA, including both choice RT and PANAS, in at least 50% (7/14) of assessments. Finally, we excluded EMA data when choice RT accuracy was less than 50% (ie, chance).

Missing data primarily arose from missed EMA assessments. Rather than imputing missing values, we used listwise deletion at the observation level within mixed-effects models, such that only available observations contributed to each model. In this study, missingness reflected missed EMA prompts rather than partially completed surveys, meaning that entire measurement occasions were absent rather than individual items within completed assessments. For this reason, we prioritized analyzing observed EMA data using mixed-effects models rather than imputing unobserved moments. Sensitivity analyses comparing models applying stricter data-cleaning criteria showed consistent results, suggesting minimal bias introduced by this criterion. After these cleaning procedures, 99 participants were included in the analyses.

### Data Analyses

Prior to analysis, we conducted a power analysis to evaluate the sample size needed to detect WP associations between NA and RT inconsistency in multilevel models. Based on prior similar EMA studies, we anticipated effects in the small-to-moderate to moderate range [7,23]. Assuming a medium effect size (*f*²=0.15), a 2-sided significance α level of .05, and a desired power of 0.80, a sample of approximately 80 to 90 participants with repeated observations was estimated to be sufficient to detect the primary WP effect. The final analytic sample included 99 participants contributing 1177 EMA observations, which provided adequate power to detect medium-sized WP effects. However, given early attrition and noncompliance, the study may be underpowered to detect small effects. Accordingly, nonsignificant findings should be interpreted cautiously.

Data were analyzed in RStudio Version 2023.06.0+421 using *tidyverse* for data wrangling and visualization (version 1.3.0) [49] and *lme4* for multilevel modeling (version 1.1‐26) [50]. First, we standardized DASS and MEDI neuroticism for ease of interpretation. Next, general linear mixed models (ie, multilevel models where sessions were modeled as nested within persons) were used to examine the impact of WP fluctuations in NA on RT inconsistency, accounting for time (ie, session) in the study, BP differences in NA, sex, age, neuroticism, and insomnia.

Predictors were entered in stages to quantify incremental variance explained, and intercepts were allowed to vary for each participant (ie, as random effects). In model 0, we ran an empty means, random-intercept-only model to examine the overall pattern of individual differences in RT inconsistency. In model 1, a 2-piece discontinuous model of change by describing the change before and after EMA session 3 as 2 separate linear slopes was fitted to capture the apparent breakpoint in session 3 (Multimedia Appendix 1). In model 2, NA was added at levels 1 and 2. In model 3, level 1 and cross-level interactions were added between EMA session and NA. In model 4, sex, age, neuroticism, insomnia, and response device were added at level 2. These covariates were included to account for potential differences in cognitive performance related to maturation, sex, and the device used.

Comparison of model fit across models, including an alternative model describing change as a continuous slope, suggests that model 4 fit the data best based on the likelihood ratio test. The diagnosis of model assumptions (linearity, constant and normal distribution of variance, and independence of residuals across levels) for model 4, along with a check of collinearity and autocorrelation as well as model fit comparisons, is provided in Multimedia Appendix 1. Model 4 equations are provided below:

Level 1 (observation level)


(1) $$\begin{array}{c} \log({sdRT}_{c_{ti}})=\beta_{0i}+\beta_{1i}({Slope\;1}_{t_{i}})+\beta_{2i}{\left(Slope\;2\right)}_{t_{i}}+\beta_{3i}({NA}_{t_{i}}-{\bar{NA}}_{i})\\ +\beta_{4i}({NA}_{t_{i}}-{\bar{NA}}_{i})\times {\left(Slope\;1\right)}_{t_{i}}+\beta_{5i}({NA}_{t_{i}}-{\bar{NA}}_{i})\times {\left(Slope\;2\right)}_{t_{i}}+e_{t_{i}} \end{array}$$

Level 2 (person level)


(2) $$\begin{array}{c} \text{Intercept: }\beta_{0i}=\gamma_{00}+\gamma_{01}(\text{Sex})+\gamma_{02}(\text{Age}-18)+\gamma_{03}(\text{Neuroticism}-16)\\ +\gamma_{04}(\text{Insomnia})+\gamma_{05}(\text{Device})+\gamma_{06}({\bar{\text{NA}}}_{i}-15)+U_{0i} \end{array}$$

(3) $$\text{Slope 1: }\beta_{1i}=\gamma_{10}+\gamma_{11}({\bar{\text{NA}}}_{i}-15)$$

(4) $$\text{Slope 2: }\beta_{2i}=\gamma_{20}+\gamma_{21}({\bar{\text{NA}}}_{i}-15)$$

(5) $$\text{WP NA}:\beta_{3i}\text{=}\gamma_{30}$$

(6) $$\text{WP NA by Slope 1}:\beta_{4i}\text{=}\gamma_{40}$$

(7) $$\text{WP NA by Slope 2}:\beta_{5i}\text{=}\gamma_{50}$$

## Results

Of the 150 recruited participants, 22.7% did not complete any EMAs and are considered early dropouts (n=34). Early dropout was defined as completing a portion of the day 1 baseline measures but not beginning EMAs over the next 13 days. The majority of early dropouts were participants from the psychology study pool.

We compared the study pool and paid study samples before and after excluding early dropouts. Before excluding early dropouts, compliance and age differed between the samples (Table 2). The study pool sample tended to be younger and less compliant. As shown in Table 2, 40% (31/78) of the student sample dropped out after day 1, compared to only 4.2% (3/72) of the paid sample. After excluding early dropouts, age no longer differed between the samples (Table 1). Sex, levels of neuroticism, depression, and anxiety did not differ between the samples (Tables1  2). This indicates that once participants began completing the study EMAs, they were not significantly more likely to be compliant if they were older or experiencing less depression and anxiety. The largest dropout occurred before beginning the EMA. Participants in the paid study were more likely to complete more than 50% of EMAs than those in the student sample. Morning assessments were chosen for data analyses to minimize potential confounds introduced by daily fatigue cycles or stress accumulation. Despite a dropout rate of 22.7% (34/150), comparisons between completers and noncompleters showed no significant differences in baseline characteristics, reducing concerns about systematic bias.

**Table 2.** Sample characteristics of participants who completed the baseline assessment (day 1; N=150)*.*

Variable	Student (n=78)	Paid (n=72)	*P* value^a^
Group, n (%)			<.001
Dropout	31 (40)	3 (4.2)	
<50	10 (13)	7 (9.7)	
>50	37 (47)	62 (86)	
Age (y; n=148), mean (SD; range)	20.0 (3.7; 18.0-47.0)	24.4 (7.9; 19.0-56.0)	<.001
Sex (n=148), n (%)	60 (77)	50 (71)	.4
Neuroticism^b^ (n=148), mean (SD; range)	16 (8; 0-34)	16 (9; 0-33)	.9
DASS-Depression^c^ (n=148), mean (SD; range)	10 (10; 0-42)	9 (9; 0-40)	.9
DASS-Anxiety^d^ (n=148), mean (SD; range)	8 (8: 0-38)	7 (7; 0-34)	.5
GAD-7^e^ total (n=148), mean (SD; range)	6.6 (5.3; 0.0-21.0)	6.1 (4.4; 0.0-19.0)	.8

a Pearson chi-square test and Wilcoxon rank-sum test.

b Multidimensional Emotional Disorder Inventory Neuroticism scale.

c DASS-Depression: Depression Anxiety and Stress Scale—Depression subscale.

d DASS-Anxiety: Depression Anxiety and Stress Scale—Anxiety subscale.

e GAD-7: Generalized Anxiety Disorder-7.

Next, we examined WP associations between NA and RT inconsistency. Results for models 1 to 4 are shown in Table 3. The intraclass correlation coefficient for RT inconsistency was 0.45 from model 0 (results not shown), indicating that 45% of its variance was due to BP mean differences. RT inconsistency decreased by 10.3% between sessions 1 and 2, but only by 1.3% after session 3 (model 1). Model 2 introduced both BP and WP NA as the main effects, while model 3 included their interaction with both slopes. Including interaction terms improved the model fit (Δ*χ*²_4_=11.58; *P*=.02).

We focus our interpretation on model 4, which examines whether RT inconsistency is predicted by WP fluctuations in NA, controlling for sex, age, insomnia level, and the response device used. Consistent with hypotheses, WP fluctuations in NA predicted RT inconsistency. Specifically, RT inconsistency increased by 2.2% when NA was higher than usual. Having a higher-than-usual NA during the first 2 sessions seems to attenuate its effect by 1.1%, but not so afterward. In addition, we observed significant main effects of our control variables: RT inconsistency was nearly 25% lower among individuals with moderate-to-severe insomnia, but using touch devices increased RT inconsistency by 11.8%. RT inconsistency was also 17% higher for women compared to men (*P*=.049).

**Table 3.** Results of general mixed-effects models predicting reaction time (RT) inconsistency^a^.

Predictors	Model 1^b^, RT inconsistency^c^			Model 2^d^, RT inconsistency			Model 3^e^, RT inconsistency			Model 4^f^, RT inconsistency		
	Estimates	95% CI	*P* value	Estimates	95% CI	*P* value	Estimates	95% CI	*P* value	Estimates	95% CI	*P* value
(Intercept)	205.253	186.422‐225.985	<.001	203.399	183.909‐224.954	<.001	184.921	164.387‐208.020	<.001	202.457	173.153‐236.721	<.001
Sessions 1‐3	0.892	0.855‐0.931	<.001	0.897	0.856‐0.939	<.001	0.944	0.893‐0.999	.08	0.930	0.879‐0.985	.03
Sessions 3‐14	0.987	0.981‐0.994	<.001	0.987	0.981‐0.994	<.001	0.986	0.979‐0.993	<.001	0.986	0.979‐0.992	<.001
morningNA^g^ bp^h^	—^i^	—	—	0.993	0.972‐1.013	.58	0.989	0.961‐1.017	.49	1.002	0.971‐1.033	.91
morningNA wp^j^	—	—	—	1.002	0.996‐1.007	.58	1.023	1.008‐1.037	.005	1.022	1.008‐1.037	.007
Sessions 1‐3 × morningNA bp	—	—	—	—	—	—	1.005	0.993‐1.018	.49	1.005	0.993‐1.018	.56
Sessions 3‐14 × morningNA bp	—	—	—	—	—	—	0.999	0.997‐1.001	.49	0.999	0.997‐1.001	.56
Sessions 1‐3 × morningNA wp	—	—	—	—	—	—	0.989	0.980‐0.999	.07	0.989	0.979‐0.998	.047
Sessions 3‐14 × morningNA wp	—	—	—	—	—	—	0.999	0.997‐1.001	.53	1.000	0.998‐1.002	.74
Male	—	—	—	—	—	—	—	—	—	0.824	0.694‐0.977	.049
Age^k^	—	—	—	—	—	—	—	—	—	1.004	0.994‐1.014	.56
Neuroticism^l^	—	—	—	—	—	—	—	—	—	1.003	0.993‐1.013	.66
Mild insomnia	—	—	—	—	—	—	—	—	—	0.912	0.786‐1.059	.38
Moderate-to-severe insomnia	—	—	—	—	—	—	—	—	—	0.757	0.622‐0.921	.02
Touch device	—	—	—	—	—	—	—	—	—	1.118	1.045‐1.195	.006

a Estimates are exponentially transformed, and *P* values are adjusted for false discovery rate.

b Random effects: residual variance (σ2): 0.14; random-intercept variance (τ00): 0.12 id (participant-level grouping variable for the random effect); intraclass correlation: 0.45; N: 99 id; observations: 1177; marginal R2/conditional R2: 0.037/0.467.

c RT inconsistency: standard deviation in reaction time (log-transformed).

d Random effects: σ2: 0.14; τ00: 0.12 id; intraclass correlation: 0.45; N: 99 id; observations: 1177; marginal R2/conditional R2: 0.039/0.469.

e Random effects: σ2: 0.14; τ00: 0.11 id; intraclass correlation: 0.45; N: 99 id; observations: 1177; marginal R2/conditional R2: 0.044/0.471.

f Random effects: σ2: 0.14; τ00: 0.10 id; intraclass correlation: 0.42; N: 99 id; observations: 1177; marginal R2/conditional R2: 0.115/0.483.

g NA: negative affect.

h BP: between-person centered at 15).

i Not applicable.

j WP: within-person.

k Age: centered at 18.

l Neuroticism: Multidimensional Emotional Disorder Inventory Neuroticism subscale centered at 16.

## Discussion

### Principal Findings

Attention dysregulation is a cardinal symptom of emotional disorders [51-53], and prior work demonstrates that trial-level RT variability reflects attentional control processes and underlying information processing efficiency [12,23,24,34]. Using a 14-day EMA with repeated cognitive (choice RT) and affective (PANAS) measures, the current study examined whether WP fluctuations in NA (deviations from an individual’s mean NA) were associated with RT inconsistency in daily life. This approach allowed for an examination of the dynamic associations between cognition and affect as they unfold in naturalistic settings.

Consistent with our primary hypothesis, momentary WP fluctuations in NA predicted greater RT inconsistency, even after accounting for BP differences in NA, neuroticism, insomnia, age, sex, and device type. Additionally, individual differences in sex and insomnia severity were associated with RT inconsistency, partially supporting our secondary hypothesis. Together, these findings suggest that acute increases in NA relative to an individual’s typical affect may disrupt attention and information processing above and beyond sleep problems or individual differences in emotional distress. These results extend prior work linking depression, anxiety [54,55], and stress [56-58] to cognitive complaints in young adults by showing that NA changes influence cognition at the momentary, WP level. Notably, the association between NA fluctuations and RT inconsistency was strongest during the early EMA sessions, suggesting that momentary affective states may be particularly disruptive to cognitive performance before participants become familiar and comfortable with a task.

### Potential Mechanisms and Interpretation

There are several potential mechanisms that account for the association between momentary NA fluctuations and RT inconsistency. Much like anxiety’s effects on performance [59], it is possible that the effects of NA on cognition depend on perceptions of task effort and difficulty, which may be influenced by practice. Early in the study, elevated NA fluctuations may more strongly tax attentional resources, whereas repeated exposure to the same tasks may reduce cognitive load. This is speculative and requires further testing.

**Figure 1.**
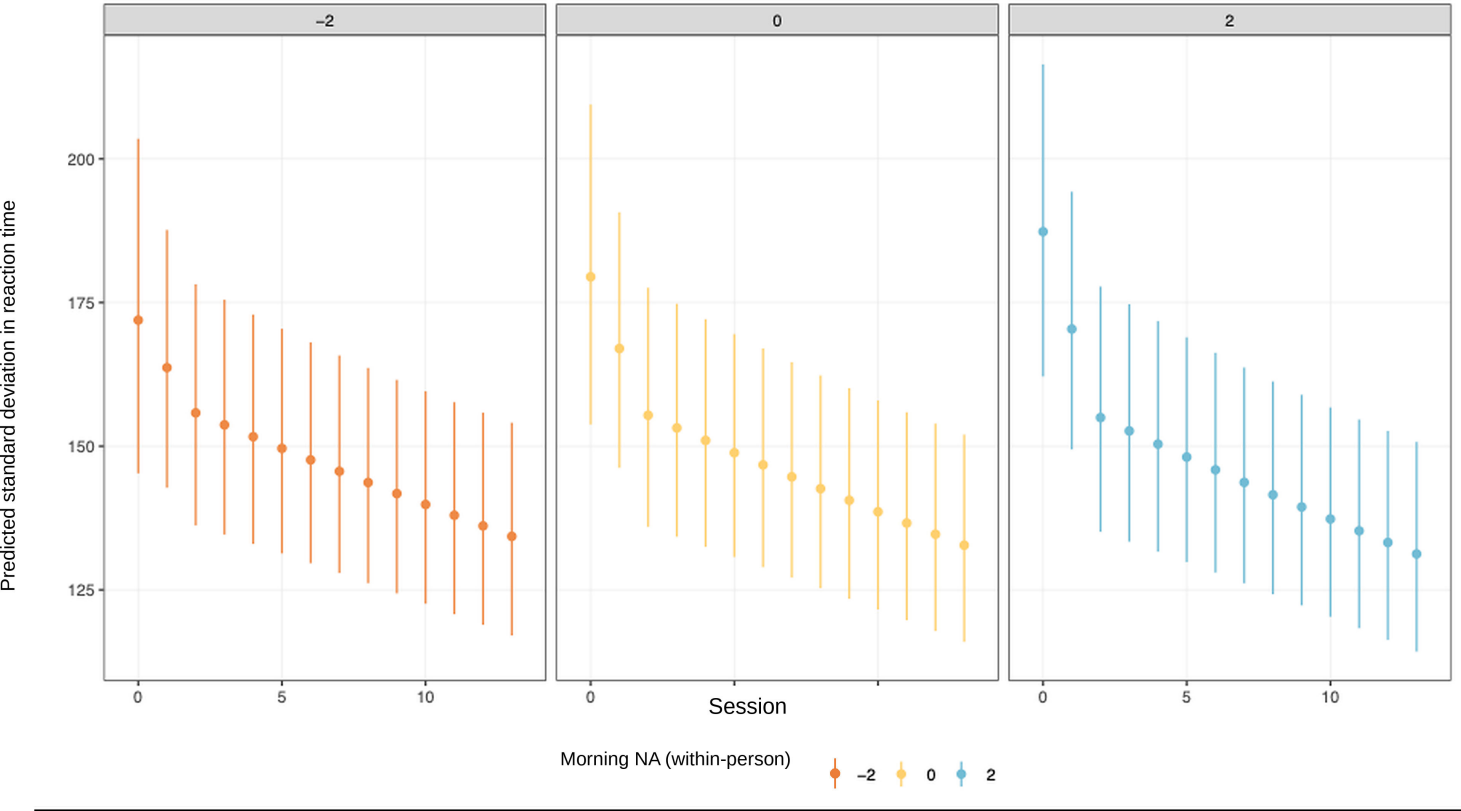
Predicted values of standard deviation reaction time (centered) based on within-person NA and session. NA: negative affect.

An alternative explanation involves speed-accuracy tradeoffs. For example, at the beginning of the study, participants experiencing low and high NA may similarly prioritize accuracy. At the end of the study, participants experiencing high NA may be more likely to prioritize speed by rapidly tapping the screen, thereby reducing RT inconsistency [60-63]. Indeed, the interaction between earlier sessions and NA, while statistically significant, highlights the dynamic nature of cognitive performance over time. An interaction plot (Figure 1) illustrates how RT inconsistency changes across sessions at varying levels of NA, providing insights into the moderating role of practice. Future research is required to replicate these findings, test competing explanations for the interaction between NA fluctuations and practice, and examine generalizability in relation to other cognitive domains. Additionally, future work could incorporate multiple equivalent cognitive tasks to reduce task-specific learning effects and allow for a more accurate assessment of cognitive variability.

### Strengths and Limitations

There are many strengths of the study and EMA approaches in general. EMA offers the opportunity to measure daily cognitive functioning in naturalistic environments, which can enhance our understanding of contemporaneous, WP relationships between cognitive and affective processes in clinical and community samples. In clinical assessments of cognition, there may be nuances that emerge outside the context of formal assessment, prior to the onset of a disorder. To be most useful, assessments of cognition should be easy to obtain in real-world settings at multiple points across time, but few validated tests exist. Our study used brief, repeated cognitive tasks that have been validated by our team [37,45,48,64-66] and are reliably administered on any mobile device (laptop, desktop, iPad, tablet, iPhone, and Android). Moreover, our approach used assessment at multiple timepoints, repeatedly, which allowed for measurement of intraindividual RT inconsistency across several timepoints, which is increasingly recognized as a warning sign of future cognitive decline and poorer prognosis. This approach allows for the detection of very subtle cognitive changes associated with depression and anxiety, as well as with aging-related changes in cognitive functioning.

Despite the many strengths of this study, several limitations should be considered. First, our sample was predominantly female and White, which limits generalizability. Future research should aim for greater diversity to validate these results in broader populations, including diverse age, gender, and socioeconomic groups. Our study also consisted of college-age, emerging adults [22]. The high dropout rate and reliance on psychology students as participants limit the generalizability of our findings. While dropout was partially mitigated by comparing baseline characteristics, the homogeneity of the sample restricts applicability to broader populations and reduces the sample size for analyses. In our study, paid participants showed lower dropout rates than psychology pool students. Participant fatigue and lack of strong incentives may have influenced results. Missing EMA assessments may introduce bias if missingness is systematically related to participants’ affect or cognitive performance. We did not formally test whether missingness was completely at random, which limits our ability to rule out systematic patterns of missingness. However, our sample was primarily impacted by noncompliance in the EMA, which could be addressed by more frequent and personalized reminder emails or a more thorough introduction to the study during the consent process. Participants were instructed to complete the assessments when they were not distracted, but we cannot confirm that occurred, and participants completed assessments at inconsistent timings based on their schedules. In future work, text messages may prompt more immediate responses than email reminders.

As with other EMA, a lack of control over the participants’ environment, device type, and network speed may have impacted our results [36,67]. A lack of experimental control is both a benefit and a limitation of EMA. Unfortunately, the study cannot determine if participants were actually trying their best or performing for course credit in an apathetic manner, and rely on data cleaning to ensure for data quality. Conducting EMA in real-world settings introduces variability due to environmental distractions (eg, noise and multitasking), device differences, and participant engagement. These factors, while enhancing ecological validity, may obscure the true relationship between NA and RT inconsistency. Future studies should consider standardized device protocols and environmental monitoring, as well as more self-reported focus checks.

Another important limitation of our design is that our choice RT task was repeated each day from days 2 to 13. Although our ongoing work addresses this, in this study, we did not alter stimuli in each EMA, which challenges efforts to disentangle the effects of task-specific learning (ie, practice effects) from the effects of memory for task stimuli and associated responses. Practice effects may also arise from factors that we did not control for, such as participant fatigue and task repetition. Future studies should administer multiple psychometrically equivalent cognitive tasks during EMA to mitigate the effects of memory on performance [68]. Isolating the effects of learning on cognitive performance holds future promise to inform digital risk phenotyping. For example, emerging evidence in older adults suggests that individual differences in learning rate and peak (asymptotic) cognitive performance are related to biological age and clinical impairment [69].

As a final limitation of our study, focusing solely on RT inconsistency as a measure of cognitive performance variability may be too narrow. Cognitive variability can be captured by various other metrics, and additional cognitive tasks can provide a more comprehensive assessment of cognitive functioning over time. We chose RT inconsistency because it is rapid and inexpensive [34] compared to a full cognitive battery. Given the push toward more individualized care through the use of basic and clinical science [70], RT inconsistency may be a particularly useful metric, with the potential to change the way we view the connection between cognition and psychiatric symptoms.

### Implications and Future Directions

In sum, momentary WP fluctuations in NA across 14 days predicted RT inconsistency, but the magnitude of this effect changed over the course of the study. These findings support the notion that NA is a form of cognitive load that disrupts cognitive performance, and even subtle fluctuations in NA are a form of stress that impacts cognitive functioning. These findings have implications for the mental health and academic performance of college students, given that internalizing conditions are on the rise and college students are increasingly seeking treatment for emotional disorders and attentional complaints. Perhaps interventions to target NA (enhancing PA, reducing NA, or directly targeting fluctuations in NA) could improve cognitive functioning in college students. For example, mindfulness-based stress approaches [71] and cognitive behavioral therapy have shown promise in reducing affective instability and improving cognitive function [72]. These interventions could be tailored to college students and emerging adults, a population particularly vulnerable to emotional and cognitive challenges, to enhance resilience and cognitive performance. By linking these findings to practical strategies, our long-term goal is to provide actionable insights for researchers and clinicians.

Additional research is necessary to examine the strength of these WP associations in relation to psychological symptoms, with potential implications for digital risk screening and preventative (eg, cognitive behavioral) intervention. Indeed, changes in affect and cognition frequently precede psychological symptoms [73,74], suggesting that momentary cognitive monitoring using scalable technologies may support early risk assessment. This study’s focus on momentary NA fluctuations complements prior research on emotional inertia [10], which has been associated with prolonged emotional disturbances and reduced cognitive resilience. Future research should examine how inertia and NA fluctuations may interact to influence cognitive performance, using EMA to capture their dynamic interplay in real-world settings.

Finally, EMA can help identify patterns of emotional responses that may signal the need for early interventions aimed at improving emotional regulation. While we focused on WP differences in NA variability, BP effects may modulate outcomes. Additional work examining how stable traits, such as neuroticism, insomnia, and sex, interact with WP fluctuations in NA to influence cognitive variability would deepen our understanding of these dynamic relationships. Future directions include the integration of passive data to better understand cognition, stress, and sleep, as well as testing in clinical samples and aging samples to understand connections between depression, cognitive impairment, and cognitive decline. Moreover, longer (eg, longitudinal burst) time series data are recommended to enable personalized risk modeling.

### Conclusions

This study provides preliminary evidence that momentary WP fluctuations in NA are associated with RT inconsistency in daily life, although the magnitude of this association changed over the course of the study. Our findings support the value of EMA and brief cognitive tasks to capture dynamic links between affect and cognitive functioning in real-world contexts. Future work using longer monitoring periods and passive data may further clarify how daily fluctuations in affect contribute to cognitive performance variability and psychiatric risk.

## Supplementary material

10.2196/64397 Multimedia Appendix 1 Supplemental materials.

## Abbreviations

BP: between-person
DASS: Depression Anxiety and Stress Scales
EMA: ecological momentary assessment
MEDI: Multidimensional Emotional Disorder Inventory
NA: negative affect
PA: positive affect
PANAS-SF: Positive and Negative Affect Schedule—Short Form
RT: reaction time
WP: within-person

## References

[1] Greenberg PE, Fournier AA, Sisitsky T, Pike CT, Kessler RC (2015). The economic burden of adults with major depressive disorder in the United States (2005 and 2010). J Clin Psychiatry.

[2] Murray CJL, Vos T, Lozano R (2012). Disability-adjusted life years (DALYs) for 291 diseases and injuries in 21 regions, 1990-2010: a systematic analysis for the Global Burden of Disease Study 2010. Lancet.

[3] Barlow DH, Ellard KK, Sauer-Zavala S, Bullis JR, Carl JR (2014). The origins of neuroticism. Perspect Psychol Sci.

[4] Crow AJD (2019). Associations between neuroticism and executive function outcomes: response inhibition and sustained attention on a continuous performance test. Percept Mot Skills.

[5] Forgas JP (2008). Affect and cognition. Perspect Psychol Sci.

[6] Dejonckheere E, Mestdagh M, Houben M (2018). The bipolarity of affect and depressive symptoms. J Pers Soc Psychol.

[7] Houben M, Van Den Noortgate W, Kuppens P (2015). The relation between short-term emotion dynamics and psychological well-being: a meta-analysis. Psychol Bull.

[8] Nelson J, Klumparendt A, Doebler P, Ehring T (2020). Everyday emotional dynamics in major depression. Emotion.

[9] Dhillon S, Videla-Nash G, Foussias G, Segal ZV, Zakzanis KK (2020). On the nature of objective and perceived cognitive impairments in depressive symptoms and real-world functioning in young adults. Psychiatry Res.

[10] Waugh CE, Kuppens P (2021). Affect Dynamics.

[11] Schoevers RA, van Borkulo CD, Lamers F (2021). Affect fluctuations examined with ecological momentary assessment in patients with current or remitted depression and anxiety disorders. Psychol Med.

[12] Jokela M (2022). Why is cognitive ability associated with psychological distress and wellbeing? Exploring psychological, biological, and social mechanisms. Pers Individ Dif.

[13] Stawski RS, MacDonald SWS, Brewster PWH, Munoz E, Cerino ES, Halliday DWR (2019). A comprehensive comparison of quantifications of intraindividual variability in response times: a measurement burst approach. J Gerontol B Psychol Sci Soc Sci.

[14] Bielak AAM, Cherbuin N, Bunce D, Anstey KJ (2014). Intraindividual variability is a fundamental phenomenon of aging: evidence from an 8-year longitudinal study across young, middle, and older adulthood. Dev Psychol.

[15] Hultsch DF, MacDonald SWS, Dixon RA (2002). Variability in reaction time performance of younger and older adults. J Gerontol B Psychol Sci Soc Sci.

[16] Kochan NA, Bunce D, Pont S, Crawford JD, Brodaty H, Sachdev PS (2016). Reaction time measures predict incident dementia in community-living older adults: the Sydney Memory and Ageing Study. Am J Geriatr Psychiatry.

[17] MacDonald SWS, Stawski RS, Diehl M, Hooker K, Sliwinski MJ (2015). Handbook of Intraindividual Variability across the Life Span.

[18] Diehl M, Hooker K, Sliwinski MJ (2015). Handbook of Intraindividual Variability Across the Life Span.

[19] MacDonald SWS, Stawski RS (2020). Longitudinal changes in response time mean and inconsistency exhibit predictive dissociations for risk of cognitive impairment. Neuropsychology.

[20] Hunt J, Eisenberg D (2010). Mental health problems and help-seeking behavior among college students. J Adolesc Health.

[21] Polanczyk GV, Willcutt EG, Salum GA, Kieling C, Rohde LA (2014). ADHD prevalence estimates across three decades: an updated systematic review and meta-regression analysis. Int J Epidemiol.

[22] Arnett JJ, Žukauskienė R, Sugimura K (2014). The new life stage of emerging adulthood at ages 18-29 years: implications for mental health. Lancet Psychiatry.

[23] Sliwinski MJ, Smyth JM, Hofer SM, Stawski RS (2006). Intraindividual coupling of daily stress and cognition. Psychol Aging.

[24] Kahneman D, Tversky A (1973). On the psychology of prediction. Psychol Rev.

[25] Blanchette I, Richards A (2010). The influence of affect on higher level cognition: a review of research on interpretation, judgement, decision making and reasoning. Cogn Emot.

[26] Shackman AJ, Salomons TV, Slagter HA, Fox AS, Winter JJ, Davidson RJ (2011). The integration of negative affect, pain and cognitive control in the cingulate cortex. Nat Rev Neurosci.

[27] Pechtel P, Pizzagalli DA (2011). Effects of early life stress on cognitive and affective function: an integrated review of human literature. Psychopharmacology (Berl).

[28] Sauer-Zavala S, Boswell JF, Gallagher MW, Bentley KH, Ametaj A, Barlow DH (2012). The role of negative affectivity and negative reactivity to emotions in predicting outcomes in the unified protocol for the transdiagnostic treatment of emotional disorders. Behav Res Ther.

[29] Rutter LA, Ten Thij M, Lorenzo-Luaces L, Valdez D, Bollen J (2024). Negative affect variability differs between anxiety and depression on social media. PLoS One.

[30] Sperry SH, Walsh MA, Kwapil TR (2020). Emotion dynamics concurrently and prospectively predict mood psychopathology. J Affect Disord.

[31] Wichers M, Peeters F, Geschwind N (2010). Unveiling patterns of affective responses in daily life may improve outcome prediction in depression: a momentary assessment study. J Affect Disord.

[32] Zainal NH, Newman MG (2021). Larger increase in trait negative affect is associated with greater future cognitive decline and vice versa across 23 years. Depress Anxiety.

[33] MacDonald SWS, Li SC, Bäckman L (2009). Neural underpinnings of within-person variability in cognitive functioning. Psychol Aging.

[34] Rutter LA, Vahia IV, Passell E, Forester BP, Germine L (2021). The role of intraindividual cognitive variability in posttraumatic stress syndromes and cognitive aging: a literature search and proposed research agenda. Int Psychogeriatr.

[35] Shiffman S, Stone AA, Hufford MR (2008). Ecological momentary assessment. Annu Rev Clin Psychol.

[36] Passell E, Strong RW, Rutter LA (2021). Cognitive test scores vary with choice of personal digital device. Behav Res Methods.

[37] Germine L, Nakayama K, Duchaine BC, Chabris CF, Chatterjee G, Wilmer JB (2012). Is the Web as good as the lab? Comparable performance from Web and lab in cognitive/perceptual experiments. Psychon Bull Rev.

[38] Rutter LA, Lind C, Howard J, Lakhan P, Germine L (2022). Posttraumatic stress symptom severity is associated with impaired processing of emotional faces in a large international sample. J Trauma Stress.

[39] Watson D, Clark LA, Tellegen A (1988). Development and validation of brief measures of positive and negative affect: the PANAS scales. J Pers Soc Psychol.

[40] Bastien CH, Vallières A, Morin CM (2001). Validation of the Insomnia Severity Index as an outcome measure for insomnia research. Sleep Med.

[41] Blais FC, Gendron L, Mimeault V, Morin CM (1997). Evaluation de l’insomnie: validation de trois questionnaires [Article in French]. Encephale.

[42] Morin CM, Belleville G, Bélanger L, Ivers H (2011). The Insomnia Severity Index: psychometric indicators to detect insomnia cases and evaluate treatment response. Sleep.

[43] Lovibond PF, Lovibond SH (1995). The structure of negative emotional states: comparison of the Depression Anxiety Stress Scales (DASS) with the Beck Depression and Anxiety Inventories. Behav Res Ther.

[44] Rosellini AJ, Brown TA (2019). The Multidimensional Emotional Disorder Inventory (MEDI): assessing transdiagnostic dimensions to validate a profile approach to emotional disorder classification. Psychol Assess.

[45] Germine LT, Joormann J, Passell E (2022). Neurocognition after motor vehicle collision and adverse post-traumatic neuropsychiatric sequelae within 8 weeks: initial findings from the AURORA study. J Affect Disord.

[46] Rutter LA, Vahia IV, Forester BP, Ressler KJ, Germine L (2020). Heterogeneous indicators of cognitive performance and performance variability across the lifespan. Front Aging Neurosci.

[47] Maljkovic V, Nakayama K (1994). Priming of pop-out: I. Role of features. Mem Cognit.

[48] Singh S, Strong R, Xu I (2023). Ecological momentary assessment of cognition in clinical and community samples: reliability and validity study. J Med Internet Res.

[49] Wickham H, Averick M, Bryan J (2019). Welcome to the Tidyverse. J Open Source Softw.

[50] Bates D, Mächler M, Bolker B, Walker S (2015). Fitting linear mixed-effects models using lme4. J Stat Softw.

[51] Dalgleish T, Watts FN (1990). Biases of attention and memory in disorders of anxiety and depression. Clin Psychol Rev.

[52] Kircanski K, Joormann J, Gotlib IH (2012). Cognitive aspects of depression. Wiley Interdiscip Rev Cogn Sci.

[53] Reinholdt-Dunne ML, Mogg K, Bradley BP (2013). Attention control: relationships between self-report and behavioural measures, and symptoms of anxiety and depression. Cogn Emot.

[54] Bierman EJM, Comijs HC, Jonker C, Beekman ATF (2007). Symptoms of anxiety and depression in the course of cognitive decline. Dement Geriatr Cogn Disord.

[55] Suddell S, Mahedy L, Skirrow C, Penton-Voak IS, Munafò MR, Wootton RE (2023). Cognitive functioning in anxiety and depression: results from the ALSPAC cohort. R Soc Open Sci.

[56] Boals A, Banks JB (2012). Effects of traumatic stress and perceived stress on everyday cognitive functioning. Cogn Emot.

[57] Klein K, Barnes D (1994). The relationship of life stress to problem solving: task complexity and individual differences. Soc Cogn.

[58] Stawski RS, Sliwinski MJ, Smyth JM (2006). Stress-related cognitive interference predicts cognitive function in old age. Psychol Aging.

[59] Yerkes RM, Dodson JD (1908). The relation of strength of stimulus to rapidity of habit‐formation. J Comp Neurol Psychol.

[60] Baird BM, Le K, Lucas RE (2006). On the nature of intraindividual personality variability: reliability, validity, and associations with well-being. J Pers Soc Psychol.

[61] Eid M, Diener E (1999). Intraindividual variability in affect: reliability, validity, and personality correlates. J Pers Soc Psychol.

[62] Hallion LS, Kusmierski SN, Caulfield MK (2020). Worry alters speed-accuracy tradeoffs but does not impair sustained attention. Behav Res Ther.

[63] Mestdagh M, Pe M, Pestman W, Verdonck S, Kuppens P, Tuerlinckx F (2018). Sidelining the mean: the relative variability index as a generic mean-corrected variability measure for bounded variables. Psychol Methods.

[64] Germine LT, Duchaine B, Nakayama K (2011). Where cognitive development and aging meet: face learning ability peaks after age 30. Cognition.

[65] Germine L, Reinecke K, Chaytor NS (2019). Digital neuropsychology: challenges and opportunities at the intersection of science and software. Clin Neuropsychol.

[66] Hartshorne JK, Germine LT (2015). When does cognitive functioning peak? The asynchronous rise and fall of different cognitive abilities across the life span. Psychol Sci.

[67] Passell E, Dillon DG, Baker JT (2019). Digital Cognitive Assessment: Results from the TestMyBrain NIMH Research Domain Criteria (RDoC) Field Test Battery Report. PsyArXiv.

[68] Hawks ZW, Strong R, Jung L (2023). Accurate prediction of momentary cognition from intensive longitudinal data. Biol Psychiatry Cogn Neurosci Neuroimaging.

[69] Oravecz Z, Harrington KD, Hakun JG (2022). Accounting for retest effects in cognitive testing with the Bayesian double exponential model via intensive measurement burst designs. Front Aging Neurosci.

[70] Stuss DT (2017). Individual differences in cognitive neuropsychology. Pers Individ Dif.

[71] Guendelman S, Medeiros S, Rampes H (2017). Mindfulness and emotion regulation: insights from neurobiological, psychological, and clinical studies. Front Psychol.

[72] Hofmann SG, Asnaani A, Vonk IJJ, Sawyer AT, Fang A (2012). The efficacy of cognitive behavioral therapy: a review of meta-analyses. Cognit Ther Res.

[73] Curtiss JE, Mischoulon D, Fisher LB (2023). Rising early warning signals in affect associated with future changes in depression: a dynamical systems approach. Psychol Med.

[74] Wichers M, Groot PC, Psychosystems, ESM Group, EWS Group (2016). Critical slowing down as a personalized early warning signal for depression. Psychother Psychosom.

[75] OSF.

