# Supplementary material for: Daily Negative Affect and Reaction Time Inconsistency in Emerging Adults: Ecological Momentary Assessment Study
Source: JMIR Mhealth Uhealth. 2026 Jun 9;14:e64397. doi: 10.2196/64397 (PMC13249201; doi:10.2196/64397)
Supplement: Multimedia Appendix 1 [file mhealth-v14-e64397-s001.docx]

**Supplemental Materials**

Note. The red line represents the average.

> anova(mod_null, mod_cont, mod_pw2)

refitting model(s) with ML (instead of REML)

Data: df

Models:

mod_null: log(sdRTc) ~ 1 + (1 | id)

mod_cont: log(sdRTc) ~ (1 | id) + session1

mod_pw2: log(sdRTc) ~ (1 | id) + pmin(session1, 2) + pmax(session1, 2)

npar AIC BIC logLik deviance Chisq Df Pr(>Chisq)

mod_null 3 1359.5 1374.7 -676.76 1353.5

mod_cont 4 1303.5 1323.8 -647.76 1295.5 57.994 1 2.629e-14 ***

mod_pw2 5 1286.9 1312.2 -638.44 1276.9 18.655 1 1.566e-05 ***

---

Signif. codes: 0 ‘***’ 0.001 ‘**’ 0.01 ‘*’ 0.05 ‘.’ 0.1 ‘ ’ 1

> anova(mod_pw2_main, mod_pw2_int, mod_pw2_full)

refitting model(s) with ML (instead of REML)

Data: df

Models:

mod_pw2_main: log(sdRTc) ~ (1 | id) + pmin(session1, 2) + pmax(session1, 2) + morningNA_bp + morningNA_wp

mod_pw2_int: log(sdRTc) ~ (1 | id) + pmin(session1, 2) + pmax(session1, 2) + morningNA_bp + morningNA_wp + pmin(session1, 2):morningNA_bp + pmax(session1, 2):morningNA_bp + pmin(session1, 2):morningNA_wp + pmax(session1, 2):morningNA_wp

mod_pw2_full: log(sdRTc) ~ (1 | id) + pmin(session1, 2) + pmax(session1, 2) + morningNA_bp + morningNA_wp + sex + age18 + medineur16 + isi_level3 + responseDevice + pmin(session1, 2):morningNA_bp + pmax(session1, 2):morningNA_bp + pmin(session1, 2):morningNA_wp + pmax(session1, 2):morningNA_wp

npar AIC BIC logLik deviance Chisq Df Pr(>Chisq)

mod_pw2_main 7 1290.1 1325.5 -638.03 1276.1

mod_pw2_int 11 1286.9 1342.7 -632.44 1264.9 11.185 4 0.0245658 *

mod_pw2_full 17 1274.1 1360.3 -620.03 1240.1 24.808 6 0.0003705 ***

---

Signif. codes: 0 ‘***’ 0.001 ‘**’ 0.01 ‘*’ 0.05 ‘.’ 0.1 ‘ ’ 1

Warning: Non-normality of residuals detected (P < .001).

Warning: Non-uniformity of simulated residuals detected (P< .001).

OK: No outliers detected.

- Based on the following method and threshold: cook (0.9).

- For variable: (Whole model)

Warning: Autocorrelated residuals detected (P < .001).

Possible heterogeneity bias due to following predictors: session1, morningNA_wp, responseDevice

OK: Error variance appears to be homoscedastic (p = 0.263)

# Check for Multicollinearity

Low Correlation

Term VIF VIF 95% CI Increased SE Tolerance Tolerance 95% CI

pmin(session1, 2) 2.35 [2.16, 2.58] 1.53 0.43 [0.39, 0.46]

pmax(session1, 2) 1.35 [1.26, 1.46] 1.16 0.74 [0.69, 0.79]

morningNA_bp 2.54 [2.32, 2.78] 1.59 0.39 [0.36, 0.43]

sex 1.25 [1.18, 1.35] 1.12 0.80 [0.74, 0.85]

age18 1.17 [1.11, 1.27] 1.08 0.85 [0.79, 0.90]

medineur16 1.43 [1.33, 1.54] 1.19 0.70 [0.65, 0.75]

isi_level3 1.15 [1.09, 1.25] 1.07 0.87 [0.80, 0.91]

responseDevice 1.12 [1.07, 1.21] 1.06 0.89 [0.82, 0.94]

pmin(session1, 2):morningNA_bp 2.66 [2.43, 2.92] 1.63 0.38 [0.34, 0.41]

pmax(session1, 2):morningNA_bp 1.74 [1.61, 1.89] 1.32 0.58 [0.53, 0.62]

Moderate Correlation

Term VIF VIF 95% CI Increased SE Tolerance Tolerance 95% CI

morningNA_wp 7.07 [6.37, 7.85] 2.66 0.14 [0.13, 0.16]

pmin(session1, 2):morningNA_wp 8.38 [7.54, 9.32] 2.89 0.12 [0.11, 0.13]

pmax(session1, 2):morningNA_wp 5.82 [5.25, 6.45] 2.41 0.17 [0.15, 0.19]
